# Personality Functioning Improvement during Psychotherapy Is Associated with an Enhanced Capacity for Affect Regulation in Dreams: A Preliminary Study

**DOI:** 10.3390/brainsci14050489

**Published:** 2024-05-11

**Authors:** Simon Kempe, Werner Köpp, Lutz Wittmann

**Affiliations:** Department of Psychology, International Psychoanalytic University, Stromstraße 1, 10555 Berlin, Germany

**Keywords:** personality functioning, personality change, dreaming, affect regulation, Zurich Dream Process Coding System

## Abstract

Background: Clinical case illustrations of patients with an impairment of personality functioning (IPF) have repeatedly reported that progress during psychotherapy is reflected by alterations in dream content. However, quantitative studies based on samples of psychotherapy patients are scarce. As a core component of both personality functioning and contemporary psychodynamic dream theory, the construct of affect regulation is of specific significance in this context. Aims: To test if improvement in personality functioning in the course of psychotherapy is associated with an increasing ability to regulate affects in dreams. Method: In a longitudinal design, affect regulation was compared in N = 94 unsolicited dream reports from the first vs. last third of long term psychotherapy of ten patients with initial IPF. Dream reports were transcribed from recordings of the sessions. Expert ratings of the level of personality functioning were obtained using the Scales of Psychological Capacities. The capacity for affect regulation was assessed using the Zurich Dream Process Coding System. Group differences were assessed using linear mixed models, controlling for dream length as well as the nested structure of this data set. Results: Patients demonstrated an increased capacity for affect regulation in dreams that was primarily evident in three core features: the complexity of dream elements (cf., e.g., parameter attributes, *p* = 0.024); the extent of affective involvement in the dream ego (cf., e.g., parameter subject feeling, *p* = 0.014); and the flexibility to regulate the dynamics of safety/involvement processes (*p* ≤ 0.001). This pattern was especially prominent in a subgroup (n = 7) of patients with more pronounced improvements in personality functioning. Conclusion: These findings support the hypotheses that decreasing IPF during psychotherapy is associated with increases in the capacity for affect regulation in dreams. Thus, researchers and therapists can utilize dream reports to illuminate the important aspects of treatment progress in clinical practice.

## 1. Introduction

### 1.1. Dreams during the Course of Psychotherapy

Although the functions of dreaming are not conclusively understood, a review of empirical research by Gazzillo et al. [1] shows that many researchers assume an adaptive function of REM sleep and dreaming. For instance, REM dreaming is considered to have a “quasi-therapeutic adaptive function” [2] (p. 235) or to “actively moderate mood overnight” [3] (p. 1). Moreover, working with dreams is a standard technique that is frequently applied in the daily practice of psychotherapy [4]. Empirical research supports four conceptual uses of dreams in psychotherapy. In clinical practice, working with dreams contributes to “(I) facilitate the therapeutic process, (II) facilitate patient insight and self-awareness, (III) provide clinically relevant and valuable information to therapists and (IV) provide a measure of therapeutic change” [5] (p. 255). The first three of these applications focus on the therapeutic use of dreams in psychotherapy, whereas the fourth stresses dreams as a (secondary) psychotherapy outcome measure. Several clinical case studies indicate that manifest dream content changes in parallel with therapeutic progress. Warner [6], for example, found that the dreams of patients who improved in psychotherapy changed “from being self-punitive or self-denying to allowing them some satisfactions and gratification” (p. 314), while dreams of patients with limited improvement in psychotherapy demonstrated little or no change. However, systematic sample research studies addressing dream content as an outcome parameter are scarce.

Glucksman and Kramer [7] report three studies in which changes in manifest dream reports between the beginning and the end of psychotherapy were found, suggesting clinical progress in such a way that the positive affect increased, as opposed to decreasing negative affect. Fischer and Kächele [8] examined 240 dreams of 8 patients from the first and last third of treatment, with respect to the theoretical orientation of the therapist (Freudian–/Jungian-Therapy). The results indicate an initial significant association between the treatment method and dream content (such as manifesting sexual contents (Freudian) or contents familiar from mythology (Jungian)) that diminishes as treatment progresses, supporting the hypothesis that patients became more independent of the therapist. In a qualitative study, Roesler [9] investigated 202 dreams of 15 patients with diverse types of diagnoses. In total, 5 dream patterns were identified, reflecting a continuum of self-efficacy and involvement (from an absent or threatened dream ego to social involvement). These dream patterns were found to be transformed towards more satisfying interactions of the dream ego when progress in symptom level and personality structure was reported by the therapist. Kuelz et al. [10] found that obsessive compulsive (OC) disorder themes decreased in 40 dreams of 9 OC inpatients during the first 5 days after the commencement of exposure therapy. Contrary to expectations, no increase in dream length or emotional intensity was evident. Taken together, evidence from the diverse types of studies that focus on different parameters suggest a continuity between improvement in waking mental health and dream experience during the course of psychotherapy.

Melstrom and Cartwright [11] compared laboratory-collected dreams before and after psychotherapy in ten patients and four control subjects. In contrast to the observations, e.g., those of Glucksman and Kramer [7], the negative affect (level of dream anxiety) was increased in patients with psychotherapy assessed as successful, whereas it barely changed in patients with unsuccessful psychotherapy outcomes and control subjects. The authors hypothesize that a successful psychotherapy outcome is reflected in a tolerance and capacity to deal with higher levels of dream anxiety, whereas lower levels indicate defensive coping or avoidance of dream anxiety.

In summary, previous research ascertained that dream content changes in the course of psychotherapy. However, several methodological shortcomings need to be reflected upon. Firstly, a lack of prospectively defined study endpoints results in a falsification problem: both increasing the positive [7] and negative [11] affect in dreams were interpreted as reflecting successful therapy. Moreover, several studies did not specify inclusion and exclusion criteria for sampling, thus the dream content analysis did not focus on specific mental disorders but on the general effects of psychotherapy.

### 1.2. Dreaming and Impairment of Personality Functioning

The level of personality functioning is a core criterion in a dimensional classification of personality pathology as introduced in the ICD-11 [12]. Moreover, the impairment of personality functioning (IPF) is also a key variable for capturing psychopathology across various mental disorders and symptoms [13] and can thus, be used to distinguish patients with personality disorders, mood, and anxiety disorders, and healthy controls [14]. As for the association of dream characteristics with IPF, research has typically focused on patients diagnosed with borderline personality disorder (BPD). Overall, there are very few sample research studies on the dreams of BPD patients, but a recurring finding was an increased frequency of nightmares and more negatively toned dreams [15]. As for the psychodynamic literature, a large range of clinical case studies, reviewed by Hau [16], focuses on the so-called borderline-dream. Overall, no consistent picture of typical borderline dream content emerged. Rather, two types of dreams can be differentiated. One group of case reports describes archaic forms of representation as, e.g., characterized by unintegrated rage, whereas other reports find that flat, realistic dreams are characteristic of BPD.

In addition to the quality of dream content in BPD patients, a dimension that has received attention from both research on IPF as well as dream research is the capacity of affect regulation. Affect regulation is typically defined as “the attempt to alter or control one’s mood or emotional state so as to maximize pleasant experiences and minimize unpleasant ones […]” including strategies such as “[…] cognitive techniques such as reframing and distraction, behavioral methods such as progressive relaxation and meditation, and unconscious processes such as denial and dissociation” [17], and is repeatedly found to be impaired in patients diagnosed with BPD (e.g., [18]).

With respect to dreams, affect regulation can be assessed by the Zurich Dream Process Coding System ([ZDPCS; [19,20]) quantifying processes in the dream narrative that balance the level between two opposing tendencies, the need for experience of safety, and the need for emotional involvement of the dream ego. Euler et al. [21] asked participants with different levels of personality functioning for dream reports within the frame of semi-standardized clinically interviews and investigated affect regulation in 62 collected dream reports applying the ZDPCS. Markers of the level of personality functioning were found to be related to the richness and complexity of the dream narrative. Kempe et al. [22] examined the affect regulation in 77 dreams reported by 20 patients with and without IPF in the first third of psychotherapy. Results indicated that patients with IPF have a limited capacity for affect regulation in dreams, reflected in three dimensions: (I) less complexity of the dream elements; (II) less involvement of the dream ego on the interactional level; and (III) less flexibility in the safety/involvement regulation of the dream dynamic. Taken together, these findings indicate that patients with IPF use more preventive affect avoidance strategies due to an increased need for security in dreaming. Noteworthy to Kempe et al.’s [22] results that impaired affect regulation in dreams is a central marker in the differentiation of patients with and without IPF at the baseline of psychotherapy, clinical case studies (e.g., the work presented in [23]) indicate that these parameters change in parallel with the therapeutic progress.

This study is premised on the hypothesis that an improvement in personality functioning during psychotherapy is reflected by an increasing capacity for affect regulation in dreams. Based on the differences in dream affect regulation between patients with and without IPF [22], it is expected that, as a result of successful psychotherapy, the affect regulation parameters of dreams from patients with initial IPF, will approach the levels shown by patients without initial IPF. The alterations in dream content in parallel to the therapeutic progress is expected to manifest in three dimensions as above (for details, see the Section 3). This study attempts to overcome the methodological limitations of previous approaches through an external assessment of therapeutic progress in personality functioning, a naturalistic clinical sample, clear inclusion and exclusion criteria for sampling, a standardized evaluation method for dream content, a falsifiable operationalization of the hypotheses, and the analysis of dream report series, rather than single dream reports in psychotherapy (which is required for an adequate basis of data to establish relationships with personality trait factors) [19,24].

## 2. Method

### 2.1. Procedure

Patients provided written informed consent and the research project was approved by the institutional review board of the IPU-Berlin (no. 2019-10). Patients were treated with (modified) psychoanalytic psychotherapy in an outpatient setting in a cooperating private practice by one experienced psychotherapist with standard audio or video recordings. The average therapy length was 238.3 sessions (SD = 90.4, range = 119–431). In one case, a cut off was made after 431 sessions because therapy had not been completed at the time of data analysis. In total, 96 sessions from the first and last third of psychotherapy, containing a dream report, were identified, based on the therapeutic documentation. Of these, eleven sessions (11.5%) were accidentally not recorded and, therefore, dreams were obtained from the therapeutic transcript made during the session.

### 2.2. Sample

Ten patients with IPF at the beginning of treatment were included into this study. Eight of these are identical with the sample in Kempe et al. [22], while two of these patients did not report dreams at the end of psychotherapy and were, therefore, replaced. The average age of the sample is 32.5 years (SD = 7.8, range = 24–47) at the time of contact (female sex = 90.0%). Inclusion criteria are an ICD-10 F60 diagnosis or a social behavior disorder (F91.2, n = 1) and a low level of personality functioning (see Section 3). On average, patients received 2.5 diagnoses (range = 1–5) according to the ICD-10 (see Table 1). In addition to psychotherapy, four patients received psychopharmacological treatment (1. patient: Fluoxetine, Amitriptyline, Methylphenidate, Candesartan, 2. patient: Escitalopram, 3. patient: St. John’s Wort, 4. patient: Quetiapine).

## 3. Measures

### 3.1. Scales of Psychological Capacities (SPC; [25])

The SPC are an expert-rated assessment tool to quantify personality functioning on the basis of 17 scales (35 subscales in total), such as self-coherence or impulse regulation. Each scale captures the severity of the respective impairment and coping possibilities to handle stressors alone (level 1), with help (level 2), or unable to, despite help (level 3), with one intermediate step each (7-point scale). Therapeutic changes in personality functioning can validly be assessed by the SPC [26]. In its original form, SPC ratings are based on semi-standardized interviews. In this study, SPC were rated based on the recording of the first and last two therapy sessions each. The construct validity and inter-rater reliability (IRR) of this procedure were satisfactory [22].

### 3.2. Zurich Dream Process Coding System (ZDPCS; [19])

The ZDPCS assesses affect regulation in dreams by analyzing strategies and capacities for regulating the course of dreams. An extensive introduction into the ZDPCS, including a fully coded dream example, as well as a comparison of the ZDPCS to other analytic approaches to dream content can be found in Kempe et al.’s work [27]. Previously, the ZDPCS has been applied for the characterization of dream affect regulation in veterans with a diagnosis of post-traumatic stress or adjustment disorder [28], as well as manifestations of psychotherapeutic progress in dreams [23]. The ZDPCS is based on a dream generation theory that extends psychoanalytic dream theory with contributions from cognitive science and artificial intelligence research. Following French [29] in applying a problem-solving paradigm, the function of a dream is understood as an attempt to solve or adapt to a complex. Complexes are defined as representations of interpersonal experiences associated with strong anxiety or disappointment which has not been disaffectualized during the process of memory consolidation [30]. Complexes originating from long-term memory are supposed to be brought to a solution by transforming the stored affective complex information back into simulated relational reality within the dream. Thereby, the dream ego is caught between the need to have a good enough sense of security (safety principle) so that the current tolerable degree of involvement of the dream ego in interpersonal processes is not exceeded, as well as the need to recommit to interpersonal relational reality (involvement principle).

### 3.3. Coding System

The aim of dream coding using the ZDPCS is to trace the affect regulatory mechanisms over the course of a dream. The dynamic shifts of the dream process between the poles of involvement and safety throughout the dream plot can be depicted. Dream reports are firstly edited (translation into presence, the deletion of comments) and then segmented. In each segment (comparable to a screenplay for the dreamwork), dream elements are coded in 5 fields with coding options in 11 main categories and 161 subcodes [28]. For this study, three ZDPCS dimensions were examined, which significantly differed between patients with and without IPF at psychotherapy baseline [22] and can thus, be assumed to be central markers for the level of personality functioning in dreams. The full scoring system, as well as its theoretical background, can be found in Moser and Hortig [19]. The IRR of the ZDPCS was examined twice [22,28] and was found to be substantial to strong (κ-range = 0.70–0.85), according to Landis and Koch [31].

### 3.4. Dream Parameters

(I) The complexity of the dream elements: Dream length (the word count of edited reports and the number of segments) is assumed to reflect the specific dimensions of personality, the ability to simulate complex inner-psychic processes, and the capacity for introspection. Furthermore, it is mandatory to adjust for dream length when comparing other dream content parameters for differences between two conditions [32]. A complex dream narrative is reflected primarily at the visual–pictorial level in the position field (PF), registering all elements, plus their attributes. A comprehensive PF is an indicator of the multifariousness of the cognitive and affective representations of the dream complex. Specific subgroups, such as the numbers of attributes in the PF, are of particular interest by highlighting how multifaceted dream elements are displayed in the dream narrative.

(II) The involvement of the dream ego on an interactional level: The frequency and the quality of interactions are registered in the interaction field (IAF) and are assumed to represent the current tolerable degree of involvement of the dream ego in interpersonal processes. A comprehensive IAF is an indicator of strong involvement of the dream ego. In addition, a categorization is made between six levels which reflect an increasing affectualization. The level of affectualization, i.e., the highest point of affectualization in a dream report, can be determined, as well as the frequencies of the different forms of interactions [20].

(III) Flexibility in the safety/the involvement regulation of the dream dynamic: The regulation between the two central tendencies in dream reports, according to the ZDPCS, is assessed. Transformations within the dream dynamic are tracked from segment to segment and differentiated with respect to whether the affectualization of the dream is increased (involvement principle) or reduced (safety principle). More frequent changes between the two poles are assumed to reflect a higher capacity in affect regulation.

A detailed description of the parameters utilized in the present study can be found in the Supplemental Materials.

## 4. Data Analysis

Statistical analyses were performed using the jamovi statistical software (v2.0). An outlier analysis of dream length (word count) resulted in excluding two dreams that were more than three standard deviations above the mean score. Due to the naturalistic study design, the number of dreams differ between patients, and the dreams of the individual patients are not independent of each other (nested data). As pointed out by Schredl [33], mixed-model approaches are mandatory in order to consider this specific nested data structure of multiple dream reports. Therefore, linear mixed models are computed to test for differences in continuous parameters after adjusting for dream length. Random effects are defined by the patient variable. Fixed effects are time points (dreams at the beginning/end of psychotherapy), dream length (word count), and the interaction term of these two. The dream length is centered in the mixed model analysis to reduce multicollinearity when computing the interaction term. For the ordinal parameters, ordinal logistic regression is required. This, however, does not allow for modeling cluster-level variables and random effects. The Shapiro–Wilks test for normality was run on each parameter and was found to be significant several times. Thus, results should be interpreted with caution, although some deviations from this normality assumption seem to be uncritical [34]. All hypotheses were tested unilaterally at *p* < 0.05 according to their clear unidirectional formulation. An inspection of patterns of change, with respect to personality functioning, revealed two clearly distinguishable subsamples. Post hoc analyses were performed for the larger subsample (n = 7), with more pronounced personality functioning improvement.

## 5. Results

### 5.1. Improvement in Personality Functioning and Descriptives

As reflected by the decreased SPC total scores, patients’ personality functioning was significantly improved at the end (M = 0.7, SD = 0.3) compared to the beginning of psychotherapy (M = 2.1, SD = 0.3) (Wilcoxon signed-rank test; z = −2.80, *p* (one-tailed) = 0.003). Patients reported 45 dreams during the first third and 51 dreams during the last third of the psychotherapy sessions. On average, 4.5 dreams were reported by patients in the beginning (SD = 1.4, range = 2–6) and 5.1 dreams in the end (SD = 1.1, range = 4–7).

### 5.2. Differences between Dreams at Beginning and End of Psychotherapy

Hypothesis 1 (Dreams are expected to be more complex): Dreams were significantly longer at the end of psychotherapy, reflected by the number of words per edited report (cf. Table 2). However, this difference was not reflected by a significantly elevated quantity of segments per dream (*p* = 0.074). Within the PF, significantly more attributes were found in dreams at the end of psychotherapy. As expected, the additional parameters of complexity were increased at the end of psychotherapy, but they missed statistical significance. In particular, dreams showed more overall codes in PF at the end of psychotherapy (*p* = 0.057), and more human object processors (*p* = 0.084).

**Figure 1:**
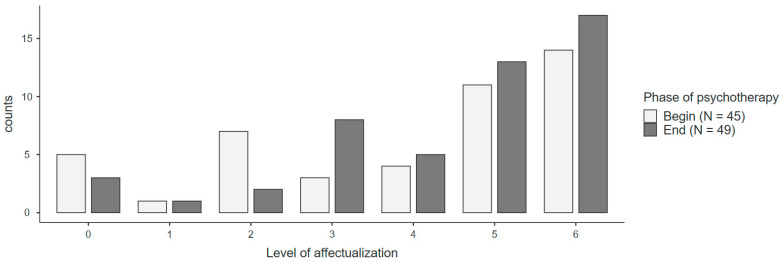
Distribution of the highest observed affectualization in dream reports (N = 10). *Note:* Level (L) 0: no interaction; L1: kinesthetic interactions; L2: displacement relations; L3: verbal relations; L4: constrained interactions; L5: resonant interactions; L6: responsive interactions.

Hypothesis 2 (Dreams are expected to show more involvement of the dream ego): The overall frequency of codes in the IAF did not significantly differ between dreams at the beginning and end of psychotherapy. Within the IAF, significantly more subject feeling was found at the end of psychotherapy. Figure 1 presents the distribution of dreams with respect to the highest observed level of affectualization (six levels) at the beginning and end of psychotherapy, according to Moser and von Zeppelin [20]. At both time points, more dreams in the high categories than in the lower categories were found. Dreams in the higher categories were more frequent at the end of psychotherapy in contrast to more dreams in lower categories at the beginning of psychotherapy, although this difference did not reach statistical significance (*p* = 0.188).

Hypothesis 3 (Dream dynamic is expected to be regulated more flexibly): Dreams at the end of psychotherapy demonstrated significantly more changes between the safety and involvement processes when compared to dreams at the beginning of psychotherapy (cf. Table 2).

### 5.3. Post Hoc Analyses

The inspection of the therapy-related improvements in personality functioning (the difference of the SPC total score from the beginning to the end of psychotherapy) revealed two subsamples: seven patients with strong effects (mean SPC change = 1.6, SD = 0.3, range = 1.2–2.0) as opposed to three patients (n = 3 reported 12 dreams each at the beginning and end of psychotherapies.) with moderate change (mean SPC change = 0.8, SD = 0.1, range = 0.7–0.9). Thus, the first group improved by 5.3 SD units with respect to the baseline values, as compared to 2.7 SD units of the second group. As previous studies (e.g., [6,11]) reported change in dreams only for successful psychotherapies, analyses were repeated for this subsample. In addition to the affect regulation parameters which changed in the overall sample, further indices, reflecting the complexity of dream elements showed significant differences, as predicted by hypothesis 1 (the quantity of segments per dream, overall codes in the position field; cf. Supplementary Table S1). Furthermore, as predicted by hypothesis 2, significantly more involvement of the dream ego (responsive interactions) was observed, as well as significant higher levels of affectualization in dreams at the end of psychotherapy (β = 0.877, *p* = 0.023, OR = 2.4, Supplementary Figure S1). The decrease in displacement relations and inanimate cognitive elements did not reach statistical significance (both *p* < 0.10).

## 6. Discussion

This analyses extends previous findings [22] of differences in affect regulation in dreams present at the baseline of psychotherapy between patients with and without IPF. Based on these findings, three specific hypotheses were formulated regarding the expected changes in dream affect regulation in response to therapy-related improvements in personality functioning. In the following section, we evaluate our hypotheses in light of the presented findings and methodological considerations.

### 6.1. Altering Dreams and Improving Personality Functioning

The results presented are largely in line with the first hypothesis that dreams are more complex at the end of psychotherapy. Dreams were significantly longer at the end of long term psychotherapy. This result contrasts with Kuelz et al. [10], who, however, examined dreams during the first five days of exposure treatment rather than long term psychotherapy, which allows for only limited comparability. The positive association between dream length and psychotherapy outcome may be explained by the enhanced ability to simulate complex inner-psychic processes. Alternatively, it could be assumed that patients described longer dreams as a result of working with dreams in psychotherapy, which could be explained by a stronger capacity for introspection or—alternatively improved dream recall. Importantly, in all other tested continuous parameters of dream content, dream length (word count) was used as a control variable, since it has been repeatedly shown that many scales correlate strongly with dream length (e.g., the work presented in [32]). Even though this effect of dream length on dream content is well known, this study is the first to methodologically adequately control for this parameter in research on therapeutic changes in dreams.

The presented results demonstrate that the complexity of dream content was increased at the end of psychotherapy. This was especially evident in significantly more attributes (ATTR) and longer dreams (word count). In addition, further parameters indicating the complexity of dream content pointed in the direction as expected: more overall codes in the position field (PF; *p* = 0.057), i.e., more equipment in the dream content and increased number of human object processors (OP; *p* = 0.084). Additionally, post hoc analyses in patients with strong personality functioning improvement showed a significant difference at the level of overall codes in the PF. The effect of significantly more ATTR and OP may be illustrated, for example, by the difference between dream content simply containing a house or a yellow house with a small balcony, round windows, and several people inside. Thus, cognitive elements in dreams at the end of psychotherapy offer more diverse affective points of contact because they are perceived as more multifaceted. Moreover, OP with a simulated inner life of their own reveal more potential to trigger affect compared to inanimate cognitive elements (CEU) [19]. An elevated number of OP at the end of psychotherapy thus, reflects an increased capacity for affect regulation. The only PF parameter which (non-significant, *p* = 0.159) decreased was the number of CEU. However, a decrease in CEU in favor of an increase in OP is in line with an increasing potential towards affect-intensive dreaming. These findings are in line with Moser and Hortig’s [19] notion that an increased capacity for affect regulation enables the dream complex to be more comprehensively represented, or, to be shaped with broad affect without overstraining the security principle. Thus, more overall codes in the PF are a central marker for increased potential for more involvement in the dreams at the end of psychotherapy.

These further results are partially in line with the second hypothesis that dreams at the end of psychotherapy display more involvement of the dream ego. While the overall frequency of codes in the IAF did not significantly differ between both time points in psychotherapy, “Subject feeling” was found to be significantly increased at the end of psychotherapy. In addition, post hoc analyses showed a significant difference with respect to “Responsive interactions” and the highest observed degree of affective involvement of the dream ego within each dream report, in line with the results of Roesler [9]. Subject feeling is coded for the experience of self-efficacy. The finding that this was increased at the end of therapy is remarkable in the context of control-mastery theory, which stresses such adaptability as a function of dreams:

“According to this model, dreams represent our unconscious attempts to find solutions to emotionally relevant problems. In dreams, people think about their main concerns, particularly those concerns that they have been unable to solve by conscious thought alone, and they try to develop and test plans and policies for dealing with them”.

[1] (p. 185)

Responsive interactions mark the distinct involvement and reciprocal regulation of interactions, which is said to be a primary marker of high affect tolerance [19]. The extension of affective intensity (according to the highest observed degree of affective involvement of the dream ego) at the end of psychotherapy is consistent with previous studies reporting higher levels of positive (e.g., the work presented in [7]) or negative affect (e.g., the work presented in [11]) in dreams as an outcome of psychotherapy. However, the ZDPCS operationalizes affective intensity by the extent of involvement and complexity of interpersonal experiences, rather than focusing on the extent of positive/negative aspects of affect in dream content analysis [27].

In accordance with the third hypothesis, dream work in patients at the end of psychotherapy was significantly more capable of oscillating between safety and involvement processes. This finding is in line with the clinical case studies of Döll-Hentschker [23], who observed a flexibilization of affect regulation in dreams in successful psychotherapies. Especially regarding patients with IPF (diagnosed with BPD), clinical case studies reported dream narratives during early stages of treatment to be simple and direct, without sudden shifts in contrast to an enrichment of the dream narratives in later stages [35], which is underlined by the present results.

In summary, the results show that, parallel to the improvement in personality functioning, the dream content approaches the parameters of patients without IPF at baseline of psychotherapy [22]. This development was visible in the overall sample, but more pronounced in the subsample (n = 7) with strong personality functioning improvement. In these successful psychotherapies (as indicated by SPC total scores changing from pre-treatment (M = 2.2, SD = 0.3) to post-treatment (M = 0.6, SD = 0.1)), the improvement in personality functioning corresponds to the patients group without IPF at baseline of psychotherapy (M = 0.6, SD = 0.1) [22] and is comparable to SPC-post-treatment-scores after psychoanalytic psychotherapy from another study (M = 0.60, SD = 0.25; [26]). The difference in therapy-related improvements in personality functioning between the two subsamples may be framed according to Kernberg’s [36] concept of personality organization. According to this model, personality pathology is assessed on a continuum, ranging from a low to high level of borderline personality organization (BPO). The sample studied here with initial IPF, i.e., severe personality pathology (N = 10), can be located at a low level of BPO. Therefore, it is plausible that therapy-related improvements in personality functioning varied, enabling patients to achieve normal/neurotic personality organization (n = 7) or high BPO level (n = 3). Accordingly, dream parameters of the latter group do not display characteristics as known from samples without IPF [22].

Taken together, the results indicate an inter-relationship between personality functioning and affect regulation in dreams. Central markers of affect regulation in dreaming related to higher levels of personality functioning are: (I) how rich and multifaceted the dream equipment is shaped, (II) to what extent affective involvement can be permitted, (III) how flexibly the dream work can oscillate in the dynamics of safety/involvement regulation.

### 6.2. Implications for Clinical Practice and Methodological Aspects

The alteration in dream-inherent affect regulation as a function of therapy-related improvement in personality functioning has implications for clinical practice and research. As summarized above, changes in dream content have been suggested several times as an outcome measure for therapeutic improvement with regard to particular factors. This study stresses that the capacity for affect regulation in dreams could serve as an outcome measure for the level of personality functioning as a criterion in a dimensional classification of personality pathology as introduced in the ICD-11 [12]. The ZDPCS, as a secondary endpoint in psychotherapy research, offers a variety of starting points to generate further hypotheses, e.g., on personality functioning in diverse diagnostic categories.

As indicated above, working with dreams in psychotherapy can serve to promote the therapeutic progress and patient’s ability for introspection [5]. Specifically at the beginning of psychotherapy, it has been noted several times that BPD patients are “usually reluctant or unable to give associations to a dream and to explore its meaning” [35] (p. 489). Thus, it is assumed that a lack of ability to symbolize leads to more concretistic forms of representation, which make an interpretation obsolete, because the meaning is openly revealed. Instead, the immediate communicative message and the expression of affect should be incorporated by the therapist. An approach such as the ZDPCS may help therapists to decide if a dream contains such signs of structural impairment or not, and which approach to dream interpretation, therefore, appears to be favorable. This is essential as our results show a rather large standard deviation and a range of affect regulation in dreams at both time points. In other words, dream reports of patients with IPF (and of those without) are by no means strictly homogenous.

### 6.3. Strengths and Limitations

To the best of our knowledge, this longitudinal study is the first to evaluate changes in unsolicited dream reports during long term psychotherapy in a systematic sample research approach based on standardized audio or video recordings, which has been performed rarely and in clinical case studies (e.g., the work presented in [23]). Previous studies were based on dream reports in psychotherapy documented by the attending clinician right after the session (e.g., the work presented in [8]) or in a sleep laboratory setting [11]. Furthermore, these results are particularly strengthened by a naturalistic clinical sample in which we focused on personality functioning. The data were analyzed retrospectively and thus, were not influenced by the longitudinal study design, which should be acknowledged as laboratory-based dream content is susceptible to experimenter bias or state factors such as participants’ laboratory experience [37]. In addition, therapeutic progress was assessed by external assessment. Furthermore, the content validity of this study is particularly strengthened by analyzing dream report series, rather than single dream reports in psychotherapy. Assessing dream report series rather than single dream reports, (which have been frequently conducted in previous research) is of importance as dream content intrapersonally varies. Thus, single dream reports provide an inadequate basis of data to establish relationships with personality trait factors [19,24]. Importantly, the resulting nested structure of this dataset and dream length were controlled for the first time in a study of alteration of dreams in psychotherapy.

Notwithstanding these strengths, this study is not free of limitations that need to be considered for any interpretation of our results. Firstly, on the data level of patients (N = 10), the sample size is relatively small, lowering statistical power in the hierarchical analyses for detecting small effects in N = 94 dream reports. By entering patients as a clustering variable, it was not possible to additionally include psychotropic medication or different diagnostic categories as a further level in the linear mixed-models. A larger sample would also have allowed testing for differences between the two subsamples. Secondly, data analysis was not performed blind with regard to the time points. However, satisfactory reliability checks based on randomly chosen dream reports against a blind second rater indicated that the occurrence of a severe bias due to this shortcoming appears to be improbable. Lastly, but importantly, it remains unclear if and how patients select dreams to then report them in psychotherapy.

## 7. Conclusions

This study explored the changes in dream-inherent affect regulation in ten patients during long term psychotherapy. For this purpose, personality functioning and N = 94 dreams from the beginning and end of treatment were compared. Changing dream content was primarily evident in three core features: the complexity of dream elements, the extent of affective involvement of the dream ego, and the flexibility to regulate dynamics of safety/involvement processes. In summary, this study provides support to the idea that improved personality functioning at the end of psychotherapy is associated with an enhanced capacity for affect regulation in dreams. Results imply that dream-inherent affect regulation can serve as an (secondary) outcome measure for the level of personality functioning. Therapists can utilize dream reports to decide between different approaches to working with dreams in clinical practice depending on levels of personality functioning.

## Figures and Tables

**Table 1:** ICD-10 diagnoses of participants (N = 10).

Variable	N
Mood [affective] disorders (F3)	3
Neurotic, stress-related, and somatoform disorders (F4)	5
Behavioral syndromes associated with physiological disturbances and physical factors (F5)	6
Disorders of adult personality and behavior (F6)	9
Emotionally unstable personality disorder (F60.3-)	
Aggressive (F60.30)	1
Borderline (F60.31)	4
Other specific personality disorders (F60.8)	4
Behavioral and emotional disorders with onset usually occurring in childhood and adolescence (F9)	1
≥2 diagnoses	9

*Note*: ICD-10 number refers to patients with at least one diagnosis from the respective cluster.

**Table 2:** ZDPCS dream characteristics (N = 10).

	First Part of Psychotherapy		Last Part of Psychotherapy		β (SE)	*p* ^a^
	M	SD (Range)	M	SD (Range)		
Dream length (word count)	154.7	113.8 (13–470)	193.7	115.6 (41–488)	38.0 (21.3)	0.039
Quantity of segments per dream	7.0	4.7 (1–22)	8.4	4.8 (3–23)	1.4 (0.9)	0.074
Position field	20.7	14.1 (2–59)	28.0	16.5 (4–91)	2.9 (1.8)	0.057
Human object processors	5.3	3.9 (0–19)	7.6	6.4 (0–29)	1.2 (0.9)	0.084
Inanimate cognitive elements	3.4	3.6 (0–17)	3.6	3.1 (0–14)	−0.5 (0.5)	0.159
Attributes	2.8	2.7 (0–12)	4.2	2.5 (0–14)	0.9 (0.4)	0.024
Static positioning of relations	0.4	0.7 (0–3)	0.5	0.8 (0–3)	0.1 (0.2)	0.383
Interaction field	6.4	6.1 (0–30)	7.0	5.1 (0–23)	−0.6 (1.0)	0.271
Displacement relations	0.9	1.2 (0–5)	0.8	1.0 (0–5)	−0.2 (0.2)	0.193
Responsive interactions	0.7	1.1 (0–5)	0.8	1.2 (0–5)	0.0 (0.2)	0.425
Subject feeling	0.1	0.3 (0–1)	0.3	0.6 (0–2)	0.2 (0.1)	0.014
Alternation between safety/involvement processes	5.0	3.5 (0–13)	8.4	5.7 (1–26)	2.1 (0.5)	<0.001

*Note*: ^a^ = Linear mixed model (*p*-value), ZDPCS = Zurich Dream Process Coding System.

## Data Availability

The data file can be shared on request. The participants of this study did not give written consent for their data to be shared publicly, so due to the sensitive nature of the research, supporting data are not available.

## Supplementary Materials

The following supporting information can be downloaded at: https://www.mdpi.com/article/10.3390/brainsci14050489/s1, Table S1: ZDPCS dream characteristics (n = 7). Figure S1: Distribution of the highest observed affectualization in dream reports (n = 7).
