# Supplementary material for: Personality Functioning Improvement during Psychotherapy Is Associated with an Enhanced Capacity for Affect Regulation in Dreams: A Preliminary Study
Source: Brain Sci. 2024 May 11;14(5):489. doi: 10.3390/brainsci14050489 (PMC11118223; doi:10.3390/brainsci14050489)
Supplement: Supplementary file 1 [file brainsci-14-00489-s001.zip › brainsci-2986170-supplementary.pdf]

Supplemental material

Parameters in the position field (PF)

In the PF, especially human beings, i.e., animated self-propelled processors (OP; object processor) and inanimate cognitive elements (CEU) are distinguished from each other. Animated objects indicate an affectualization of the dream complex (involvement) by already containing a latent model of the relational structure. Contrarily, inanimate objects display affect bonding (security) by not containing a model of an inherently communicative affect system. Specific attributes (ATTR) conform to a focus on the dream complex. Static positioning of relations (POS REL), i.e., linkages of cognitive elements without interaction, represent relational representations that contain little potential for change. Such processes in the PF serve to regulate potentially subsequent interactions (captured in the IAF).

Parameters in the interaction field (IAF)

In the IA, different forms of interaction are distinguished. The structure of the represented interactivity ("representations interaction generalized", RIG, Stern (1985)) can be of varying complexity (Moser & Hortig, 2019; Moser & von Zeppelin, 1996). A hierarchy of six levels of increasingly intense interactions is described below.

- 1) Kinesthetic interactions (IRC KIN; dream-ego with CEU): Relations between the dream ego and inanimate objects reflect an involvement that, compared to interpersonal relationships, demands less regulation, and can thus be controlled more strongly.
- 2) Displacement relations (IRD; the dream ego positions itself as a spectator of the event): What displacement relations have in common is namely, that the dream-ego connects with the interactive field only indirectly, through identifying itself as a spectator. Thus, these processes serve to limit the involvement of the dream ego.
- 3) Verbal relations (VR; dream-ego with object): It is assumed that verbal interpersonal communication in dreams is under stronger affective control than events on the sensory concretistic level.

- 4) Constrained interactions (IRC constr; dream ego with restricted object): Interactions with affective restricted objects such as animals or personified non-persons (for example, a talking tree) have a limited potential for affective exchange because of their asymmetrical character. The dream ego is often already affectively related, but the interaction lacks reciprocity.
- 5) Resonant interactions (IRC RES; dream-ego with human object linked by parallel behavior): Parallel interpersonal relations allow the dream ego to experience belonging, but specific wishes and motivations (by dream ego or object) that dominate the interaction remain hidden.
- 6) Responsive interactions (IRC RESP; circular interactions between dream-ego and human object): Circular interpersonal interactions require affective regulation of reciprocally related behavior. Accordingly, a shared model of the relationship is created, which indicates a high level of involvement.
- In addition, interpersonal interactions can be assessed in terms of the dream-ego experiencing self-efficacy (subject feeling). Being able to influence interpersonal interactions with regard to one's own wishes speaks to capacities of affect regulation.

Supplementary Table S1: *ZDPCS dream characteristics (n = 7)*

	First part of psychotherapy		Last part of psychotherapy		β (SE)	p [a]
	M	SD (Range)	M	SD (Range)		
Dream length (word count)	153.5	113.3 (13-470)	209.8	122.1 (45-488)	56.9 (26.2)	0.017
Quantity of segments per dream	6.5	3.9 (1-16)	8.8	4.9 (3-23)	2.3 (1.1)	0.019
Position field	20.1	13.6 (2-59)	30.2	17.2 (10-91)	4.0 (2.4)	0.048
Human object processors	5.2	4.3 (0-19)	8.4	6.7 (0-29)	1.6 (1.1)	0.071
Inanimate cognitive elements	3.4	3.5 (0-17)	3.4	2.7 (0-11)	-1.0 (0.6)	0.051
Attributes	2.6	2.3 (0-7)	4.6	2.6 (1-14)	1.4 (0.5)	0.006
Static positioning of relations	0.5	0.8 (0-3)	0.5	0.8 (0-3)	-0.2 (0.2)	0.176
Interaction field	6.0	5.3 (0-23)	7.3	5.3 (0-23)	-0.3 (1.1)	0.386
Displacement relations	0.9	1.3 (0-5)	0.8	0.8 (0-4)	-0.3 (0.2)	0.091
Responsive interactions	0.5	0.9 (0-4)	1.1	1.3 (0-5)	0.5 (0.3)	0.029
Subject feeling	0.1	0.3 (0-1)	0.3	0.6 (0-2)	0.2 (0.1)	0.023
Alternation between safety-/involvement processes	4.7	3.3 (0-13)	9.1	6.1 (1-26)	2.5 (0.7)	< .001

Note: [a] = Linear mixed model (p-value), ZDPCS = Zurich Dream Process Coding System

Supplementary Figure S1: *Distribution of the highest observed affectualization in dream reports (n = 7)*

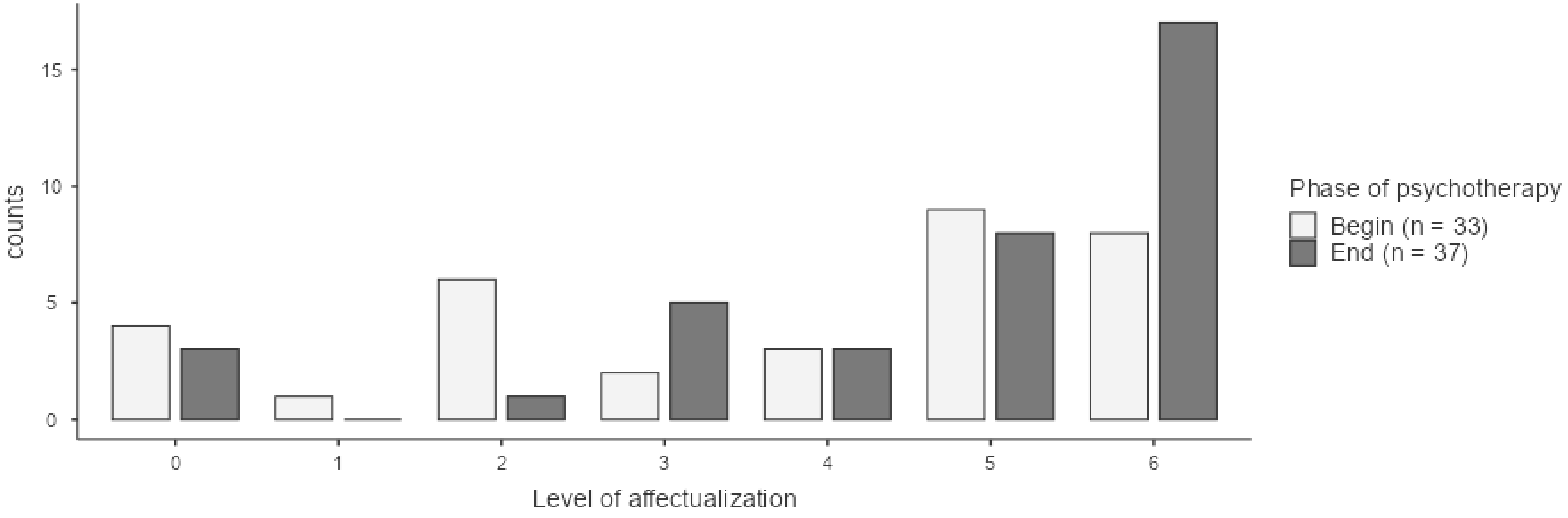

Note: Level (L) 0: no interaction; L1: kinesthetic interactions; L2: displacement relations; L3: verbal relations; L4: constrained interactions; L5: resonant interactions; L6: responsive interactions
